# A clinical prediction model for predicting the risk of liver metastasis from renal cell carcinoma based on machine learning

**DOI:** 10.3389/fendo.2022.1083569

**Published:** 2023-01-05

**Authors:** Ziye Wang, Chan Xu, Wencai Liu, Meiying Zhang, Jian’an Zou, Mingfeng Shao, Xiaowei Feng, Qinwen Yang, Wenle Li, Xiue Shi, Guangxi Zang, Chengliang Yin

**Affiliations:** ^1^ Department of Urology, The First Affiliated Hospital of Anhui University of Chinese Medicine, Hefei, China; ^2^ Clinical Medical Research Center, Xianyang Central Hospital, Xianyang, China; ^3^ Department of Orthopaedic Surgery, The First Affiliated Hospital of Nanchang University, Nanchang, China; ^4^ Department of Gastroenterology and Hepatology, Chinese People's Liberation Army (PLA) General Hospital, Beijing, China; ^5^ Department of Neuro Rehabilitation, Shaanxi Provincial Rehabilitation Hospital, Xi’an, China; ^6^ School of Computer Science and Engineering, North Minzu University, Yinchuan, China; ^7^ State Key Laboratory of Molecular Vaccinology and Molecular Diagnostics and Center for Molecular Imaging and Translational Medicine, School of Public Health, Xiamen University, Xiamen, China; ^8^ Department of Geriatrics, Shaanxi Provincial Rehabilitation Hospital, Xi’an, China; ^9^ Faculty of Medicine, Macau University of Science and Technology, Macau, Macao SAR, China

**Keywords:** renal cell carcinoma, liver metastasis, machine learning, prognostic factors, web calculator

## Abstract

**Background:**

Renal cell carcinoma (RCC) is a highly metastatic urological cancer. RCC with liver metastasis (LM) carries a dismal prognosis. The objective of this study is to develop a machine learning (ML) model that predicts the risk of RCC with LM, which is used to assist clinical treatment.

**Methods:**

The retrospective study data of 42,547 patients with RCC were extracted from the Surveillance, Epidemiology, and End Results (SEER) database. ML includes algorithmic methods and is a fast-rising field that has been widely used in the biomedical field. Logistic regression (LR), Gradient Boosting Machine (GBM), Extreme Gradient Boosting (XGB), random forest (RF), decision tree (DT), and naive Bayesian model [Naive Bayes Classifier (NBC)] were applied to develop prediction models to predict the risk of RCC with LM. The six models were 10-fold cross-validated, and the best-performing model was selected based on the area under the curve (AUC) value. A web online calculator was constructed based on the best ML model.

**Results:**

Bone metastasis, lung metastasis, grade, T stage, N stage, and tumor size were independent risk factors for the development of RCC with LM by multivariate regression analysis. In addition, the correlation of the relative proportions of the six clinical variables was shown by a heat map. In the prediction models of RCC with LM, the mean AUC of the XGB model among the six ML algorithms was 0.947. Based on the XGB model, the web calculator (https://share.streamlit.io/liuwencai4/renal_liver/main/renal_liver.py) was developed to evaluate the risk of RCC with LM.

**Conclusions:**

This XGB model has the best predictive effect on RCC with LM. The web calculator constructed based on the XGB model has great potential for clinicians to make clinical decisions and improve the prognosis of RCC patients with LM.

## 1 Introduction

Renal cell carcinoma (RCC) accounts for approximately 2% of global cancer diagnoses and deaths (1). RCC incidence rates are increasing, particularly in developed countries. The reason partially may be because of imaging, typically with a magnetic resonance imaging (MRI), computed tomography (CT) scan, or ultrasound (2, 3). RCC is the deadliest urological neoplasm and has a dismal late-stage 5-year survival rate of 12% (4, 5). Although most incidentally detected lesions are small low-grade tumors, 25%–30% of RCC patients present with distant metastasis at initial diagnosis (6, 7).

The liver is one of the common metastatic sites of RCC, with estimates of involvement in 20% of patients with metastatic RCC (8). Unfortunately, the development of liver metastasis (LM) is generally considered a poor prognostic factor and is often associated with more widespread disease (9, 10). The duration of median progression-free survival and overall survival in patients with LM was significantly shorter than that of patients without LM (11). The median overall survival of RCC patients with LM is<12 months and shorter than that in patients with metastases from other sites (e.g., lung, brain, lymph nodes, etc.) (12, 13). Moreover, metastatic tumors render patients ineligible for surgery, especially when critical organs are involved. Systemic immunotherapy has been the standard therapy for metastatic RCC (mRCC) over the past few decades (11). However, LM responds poorly to systemic therapy, with a 15% objective response rate to immunochemotherapy (14). Thus, early detection and early intervention are crucial for RCC treatment. The risk of RCC patients with LM is an urgent issue. The treatment of RCC with LM remains to be explored. New approaches and early detection are crucial for RCC treatment.

Linear regression as an important machine learning (ML) method can build a linear connection between dependent and independent variable sets to predict uncertainties. The researchers focused on predicting whether this patient is healthy or not, but that is not effective (15). A model was needed to illustrate that one person is moving toward this disease during the early detection of the disease. Artificial intelligence (AI) was implemented in the medical and health fields in recent years (15). ML is one intelligent branch of the AI field and a discipline in computer science wherein computers are programmed to process the input data. It focuses on how computers learn and improve from data. The learning algorithms create models that can make predictions or decisions without being explicitly programmed to perform the task. The function of disease diagnosis is important for its application in cancer-related diagnosis and treatment for the performance of appropriate retrospective analysis (16, 17). ML methods were used to establish a predictive model, which were tested and trained to acquire a suitable algorithmic model to quickly and accurately diagnose, predict, and monitor disease. And ML methods were helpful for the design of the treatment plan by doctors (18).

Although similar ML prediction methods were reported for RCC, there was still less research in RCC with LM (19, 20). In our study, data of 852 RCC patients from the Surveillance, Epidemiology, and End Results (SEER) database were used, and six ML models [namely, logistic regression (LR), Gradient Boosting Machine (GBM), Extreme Gradient Boosting (XGB), random forest (RF), decision tree (DT), and Naive Bayes Classifier (NBC)] were carried out. The XGB prediction model showed the best performance in predicting the risk of RCC with LM. A predictive web calculator was constructed for clinicians managing predicted risks and establishing personalized treatment strategies of RCC patients with LM.

## 2 Materials and methods

### 2.1 Patient cohorts

#### 2.1.1 The SEER cohort (training group)

The training RCC patient group’s information was extracted from the SEER database of the National Cancer Institute. SEER is one of the most representative large oncology registry databases in North America, in which patient demographics (age, gender, stage, and so on), site of the primary tumor, pathological type, method of diagnosis, treatment, time to death, and survival time were included (21, 22). Detailed information about SEER can be found on the official website (http://seer.cancer.gov/about/). The SEER database has public datasets and does not contain any sensitive content or identifying information of patients; these data can be used without ethics committee approval.

#### 2.1.2 Patient cohort (validation group)

The information data of RCC patients were obtained from the Second Affiliated Hospital of Dalian Medical University. All data collection was performed following the guidelines approved by the Second Affiliated Hospital of Dalian Medical University. The clinical information of patients in this study included marital status, gender, age, race, survival status, survival time, sequence number, primary site, laterality, grade, pathological staging, T stage, N stage, tumor size, bone metastasis, brain metastasis, LM, and lung metastasis. All cancer samples were classified in accordance with TNM staging [American Joint Committee on Cancer (AJCC)]. Pathological staging was diagnosed by at least two dedicated genitourinary pathologists.

### 2.2 Clinical data screening

SEER*stat (8.3.6) software was employed to extract the available data of the training group from a retrospective cohort study. In our study, the SEER database’s tumor nomenclature and coding manual (23) and the International Classification of Diseases tumor morphology code ICD-O-3 (24) were employed to extract the available data of 2010–2017 kidney cancer patients for the training group (25). The inclusion/exclusion criteria were as follows: 1) distinct diagnosis with pathology (validation group was diagnosed by at least two dedicated genitourinary pathologists); 2) RCC was the primary tumor; 3) integral follow-up information; 4) complete clinical characteristic factors of patients; 5) clear stage and grade; 6) survival time more than 0 month. Finally, 42,547 patients of the training group and 852 patients of the validation group were screened according to the inclusion/exclusion criteria. Information on all variables was complete for these patients.

### 2.3 Statistical analysis

The numerical variables were expressed as mean ± standard deviation (SD), and the count data were expressed as frequencies and percentages. Shapiro–Wilk test, *t*-test, chi-square test, univariate and multivariate LR analysis, Least absolute shrinkage and selection operator (LASSO) regression analysis, correlation heat map, 10-fold cross-validation plot, and AUC plot were performed using SPSS 26.0 software (SPSS Inc., Chicago, USA), R language (version 4.0.5), and Python (version 3.8). *p*< 0.05 was considered statistically significant. ML models were designed based on the scikit-learn (version 0.24) library.

### 2.4 Feature engineering and selection

Numerical variables such as tumor size were processed using data standardization methods. Category variables such as T stage were processed using label-encoding methods. The LASSO regression method was used to screen for meaningful combinations of features for predicting the risk of RCC patients with LM. Correlation analysis was used to analyze the correlation among the selected features. Feature importance analysis was performed on the variables based on the Permutation Importance principle.

### 2.5 Predictive model building and validation

Six ML models of LR, GBM, XGB, RF, DT, and NBC were used to predict the risk of RCC patients with LM (26–31). Random oversampling methods were used to deal with the imbalance in the distribution of the data. Ten-fold cross-validation was used to compare the performance of the models. Random search method was used to adjust the hyperparameters of the model. Prediction results of the model were binary output and probabilistic output. XGB is an integration algorithm based on boost. It is typical of the integration of cart tree, which is an improvement of the gradient tree boosting.


$$L(\phi )=\sum_{i}l(\hat{y},y_{i})+\sum_{k}\Omega (f_{k})\;where\;\Omega (f)=\gamma T+\frac{1}{2}\lambda \|w{\|}^{2}$$

Here, l is a differentiable convex loss function that measures the difference between the prediction $\hat{y}i$ and the target *y_i_*. The second term Ω penalizes the complexity of the model. The probabilistic output results are evaluated using the receiver operating characteristic curve (ROC). The ROC is an intuitive method for evaluating the sensitivity and specificity. The testing effect is dependent on the value of the area under the ROC (AUC); the higher the value of the AUC, the better is the effect of the ML model. A colormap was used to show the comparison between the predicted results of the models and the actual situation in the test set. The highest AUC value of one of the ML models was selected as the best prediction model. A web-based online calculator based on the prediction model was also constructed. The code for each step of the article data analysis can be found in Github; see https://github.com/chengliangyin/chengliangyin1.

## 3 Results

### 3.1 Demographic characteristics and parameter screening

**Figure 1:**
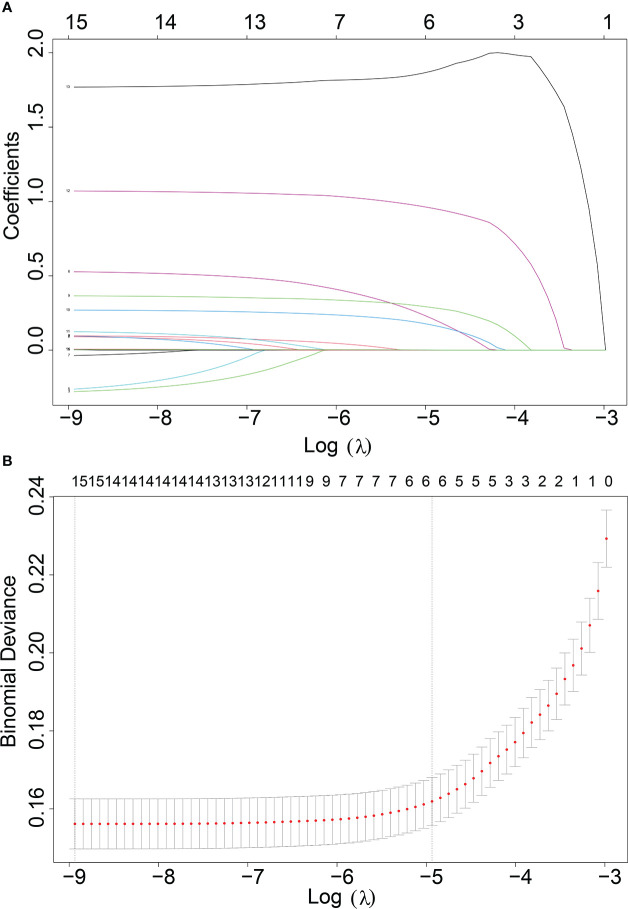
**(A)** Optimal parameter (λ) selection in the LASSO model, with the optimal tuning parameter log(λ) in the horizontal coordinate and the regression coefficients in the vertical coordinate. **(B)** Distribution of LASSO coefficients for the clinical factors, with the optimal tuning parameter log(λ) in the horizontal coordinate and the binomial deviation in the vertical coordinate.

**Figure 2:**
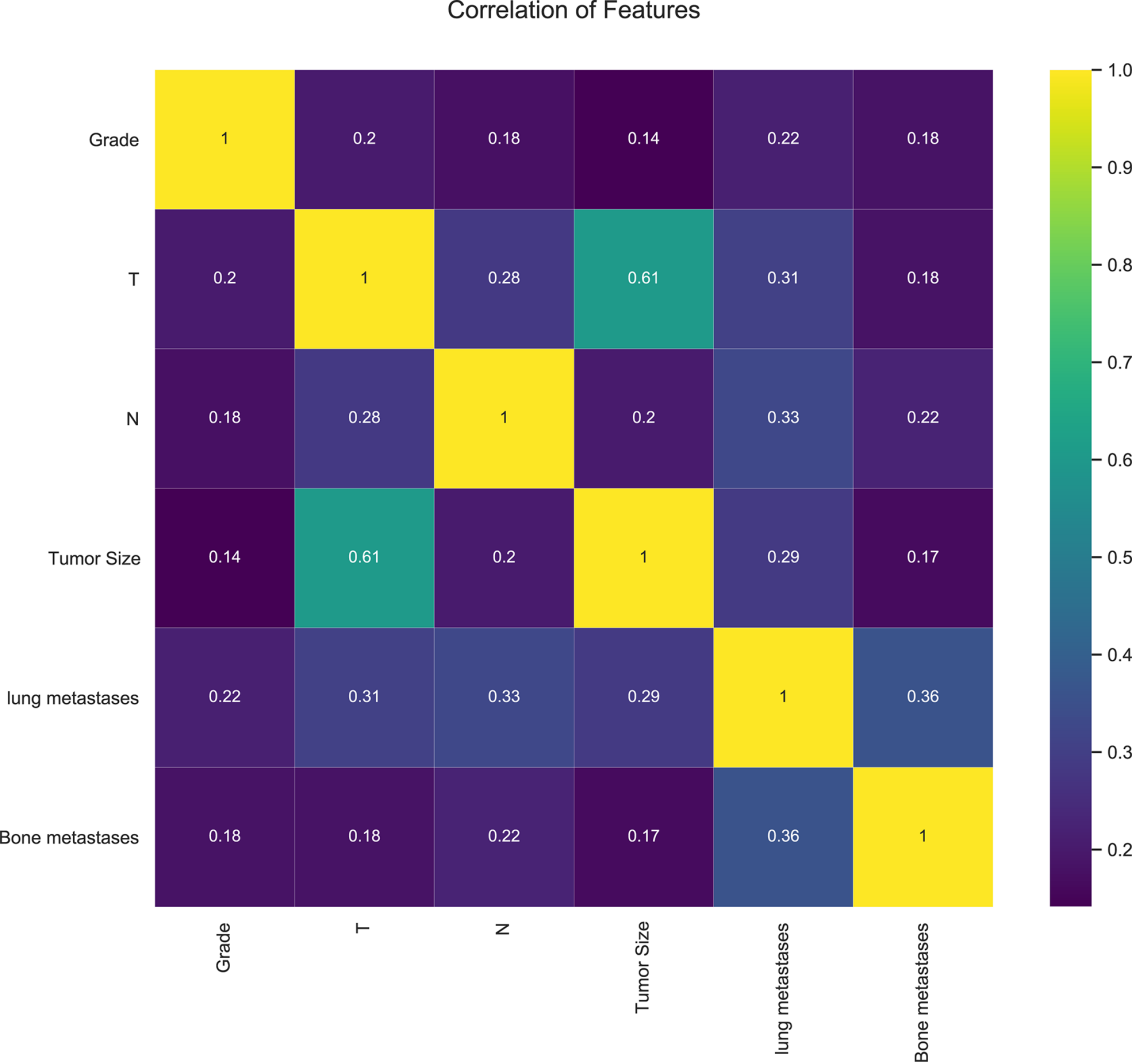
The correlation heat map of six features.

In our study, 42,547 RCC patients were included in the training group and 852 RCC patients were in the validation group. The median age of the training and validation groups was 63.49 years (SD = 13.07) and 63.87 years (SD = 13.08), respectively. The median survival time of the training group was 39.12 months (SD = 30.69), and it was 37.17 months (SD = 30.82) in the validation group. The median tumor size was 51.59 mm (SD = 41.13) in the training group and 52.07 mm (SD = 7.18) in the validation group. The *p*-values of age, sequence number, survival time, survival status, gender, tumor size, and lung metastasis were 0.403, 0.129, 0.066, 0.643, 0.646, 0.734, and 0.392 by comparing the training and validation groups. There were no statistically significant differences (all *p* > 0.05, **Table 1**). While marital status, ethnic primary site, laterality, grade, pathological staging, T stage, N stage, bone metastases, brain metastases, and LM showed statistically significant differences between the training and validation groups (all *p*< 0.05, **Table 1**). In the training group, there were 1,030 (2.4%) RCC patients LM, and there were 32 (3.8%) in the validation group (**Table 1**). The LASSO regression method was used to screen for meaningful combinations of risk factors for predicting the risk of RCC patients with LM. Six interesting parameters, namely, bone metastasis, lung metastasis, grade, T stage, N stage, and tumor size, were highly correlated with the risk of RCC patients with LM (**Figures 1A, B**). The correlation heat map demonstrated that six features were used to predict the risk of RCC patients with LM. Thus, these six features were used as predictors in the correlation heat map (**Figure 2**).

**Table 1:** Baseline patient data from the training and validation groups.

Characteristics	Level		Training group (N=42,547)	Validation group (N=852)	*p*
Marital status (%)	Married		25,009 (58.8)	560 (65.7)	<0.001
	unknown		2,116 (5.0)	0 (0.0)	
	unmarried		15,422 (36.2)	292 (34.3)	
Age [mean (SD)]	NA		63.49 (13.07)	63.87 (13.08)	0.403
Race ethnicity (%)	black		5,378 (12.6)	0 (0.0)	<0.001
	Chinese		511 (1.2)	852 (100.0)	
	other		3,394 (8.0)	0 (0.0)	
	white		33,264 (78.2)	0 (0.0)	
Sequence number (%)	more		14,008 (32.9)	259 (30.4)	0.129
	One primary only		28,539 (67.1)	593 (69.6)	
times [mean (SD)]	NA		39.12 (30.69)	37.17 (30.82)	0.066
status (%)	alive		31,486 (74.0)	624 (73.2)	0.643
	dead		11,061 (26.0)	228 (26.8)	
Sex (%)	female		15,032 (35.3)	308 (36.2)	0.646
	male		27,515 (64.7)	544 (63.8)	
Primary site (%)	C64.9-Kidney		40,466 (95.1)	762 (89.4)	<0.001
	C65.9-Renal pelvis		2,081 (4.9)	90 (10.6)	
Grade (%)	Moderately differentiated		14,646 (34.4)	313 (36.7)	<0.001
	Poorly differentiated		8,903 (20.9)	254 (29.8)	
	Undifferentiated; anaplastic		3,329 (7.8)	68 (8.0)	
	unknown		12,282 (28.9)	138 (16.2)	
	Well differentiated		3,387 (8.0)	79 (9.3)	
Laterality (%)	left		21,021 (49.4)	422 (49.5)	<0.001
	other		85 (0.2)	7 (0.8)	
	right		21,441 (50.4)	423 (49.6)	
Pathological (%)*	8120/3		1,140 (2.7)	33 (3.9)	0.005
	8130/3		1,033 (2.4)	30 (3.5)	
	8260/3		5,274 (12.4)	78 (9.2)	
	8310/3		22,588 (53.1)	470 (55.2)	
	8312/3		7,770 (18.3)	149 (17.5)	
	8317/3		2,230 (5.2)	50 (5.9)	
	other(n<1000)		2,512 (5.9)	42 (4.9)	
T (%)		T1	27,878 (65.5)	513 (60.2)	0.011
		T2	4,239 (10.0)	104 (12.2)	
		T3	8,401 (19.7)	186 (21.8)	
		T4	1,129 (2.7)	23 (2.7)	
		TX	900 (2.1)	26 (3.1)	
N (%)		N0	38,343 (90.1)	753 (88.4)	0.017
		N1	2,402 (5.6)	65 (7.6)	
		N2	198 (0.5)	0 (0.0)	
		NX	1,604 (3.8)	34 (4.0)	
Tumor size [mean (SD)]		NA	51.59 (41.13)	52.07 (37.18)	0.734
Bone metastases (%)		No	40,654 (95.6)	785 (92.1)	<0.001
		Yes	1,893 (4.4)	67 (7.9)	
Brain metastases (%)		No	42,016 (98.8)	834 (97.9)	0.037
		Yes	531 (1.2)	18 (2.1)	
Liver metastasis (%)		No	41,517 (97.6)	820 (96.2)	0.017
		Yes	1,030 (2.4)	32 (3.8)	
Lung metastases (%)		No	39,454 (92.7)	783 (91.9)	0.392
		Yes	3,093 (7.3)	69 (8.1)	

*Pathological: 8120/3 Transitional cell carcinoma, 8130/3 Papillary transitional cell carcinoma, 8260/3 Papillary (Chromophil), 8310/3 Clear Cell, 8312/3 Renal cell carcinoma, NOS, 8317/3 Chromophobe, Other: other specific renal cell carcinoma types.

### 3.2 Univariate and multivariate logistic regression analysis

Univariate and multivariate LR analyses were used to analyze the relative risk of RCC patients with LM. Univariate LR analysis showed that bone metastasis, lung metastasis, grade, T stage, N stage, and tumor size were significant risk factors of RCC patients with LM (all *p*< 0.05, **Table 2**).

**Table 2:** Univariate and multivariate logistic regression of the risk of liver metastasis in patients with renal cancer.

Characteristics	Univariate logistic regression			Multivariable logistic regression		
	OR	CI	*p*	OR	CI	*p*
Bone metastases						
No	Ref	Ref	Ref	Ref	Ref	Ref
Yes	15.61	13.62-17.89	<0.001	2.72	2.31-3.19	<0.001
Grade						
Well differentiated	Ref	Ref	Ref	Ref	Ref	Ref
Moderately differentiated	1.39	0.62-3.09	0.422	1.06	0.47-2.38	0.885
Poorly differentiated	7.77	3.64-16.61	<0.001	2.73	1.26-5.9	0.011
Undifferentiated anaplastic	17.12	7.97-36.77	<0.001	2.74	1.25-5.99	0.011
unknown	30.34	14.4-63.9	<0.001	7.31	3.43-15.55	<0.001
Lung metastases						
No	Ref	Ref	Ref	Ref	Ref	Ref
Yes	25.31	22.19-28.89	<0.001	4.88	4.17-5.71	<0.001
N						
N0	Ref	Ref	Ref	Ref	Ref	Ref
N1	16.93	14.72-19.47	<0.001	2.9	2.46-3.42	<0.001
N2	8.63	5.33-13.97	<0.001	2.23	1.28-3.89	0.005
NX	7.23	5.91-8.84	<0.001	2.05	1.61-2.61	<0.001
T						
T1	Ref	Ref	Ref	Ref	Ref	Ref
T2	6.12	4.94-7.58	<0.001	2.12	1.66-2.71	<0.001
T3	6.39	5.33-7.67	<0.001	2.69	2.17-3.34	<0.001
T4	33.34	27-41.18	<0.001	6.1	4.71-7.89	<0.001
TX	29.87	23.75-37.57	<0.001	3.73	2.84-4.9	<0.001
Tumor size	1.01	1.01-1.01	<0.001	1.00	1.00-1.00	<0.001

OR, odds ratio; CI, confidence interval.

Multivariate LR analysis has further shown that bone metastases [odds ratio (OR) = 2.72, 95% CI 2.31–3.19, *p*< 0.001], lung metastases (OR = 4.88, 95% CI 4.17–5.71, *p*< 0.001), grade (poorly differentiated OR = 2.73, 95% CI 1.26–5.9, *p*< 0.05; undifferentiated OR = 2.74, 95% CI 1.25–5.99, *p*< 0.05; unknown OR = 7.31, 95% CI 3.43–15.55, *p*< 0.001), T stage (T2 OR = 2.12, 95% CI 1.66–2.71, *p*< 0.001; T3 OR = 2.69, 95% CI 2.17–3.34, *p*< 0.001; T4 OR = 6.1, 95% CI 4.71–7.89, *p*< 0.001; Tx OR = 3.73, 95% CI 2.84–4.9, *p*< 0.001), N stage (OR = 2.9, 95% CI 2.46–3.42, *p*< 0.001; N2 OR = 2.23, 95% CI 1.28–3.89, *p*< 0.01; Nx OR = 2.05, 95% CI 1.61–2.61, *p*< 0.001), and tumor size (OR = 1.00, 95% CI 1.00–1.00, *p*< 0.0.001) were significant risk factors of RCC patients with LM.

The above results suggested that bone metastases, lung metastases, grade, T stage, N stage, and tumor size were independent risk factors of RCC patients with LM (all *p*< 0.05, **Table 2**).

### 3.3 Optimal prediction model selection

**Figure 3:**
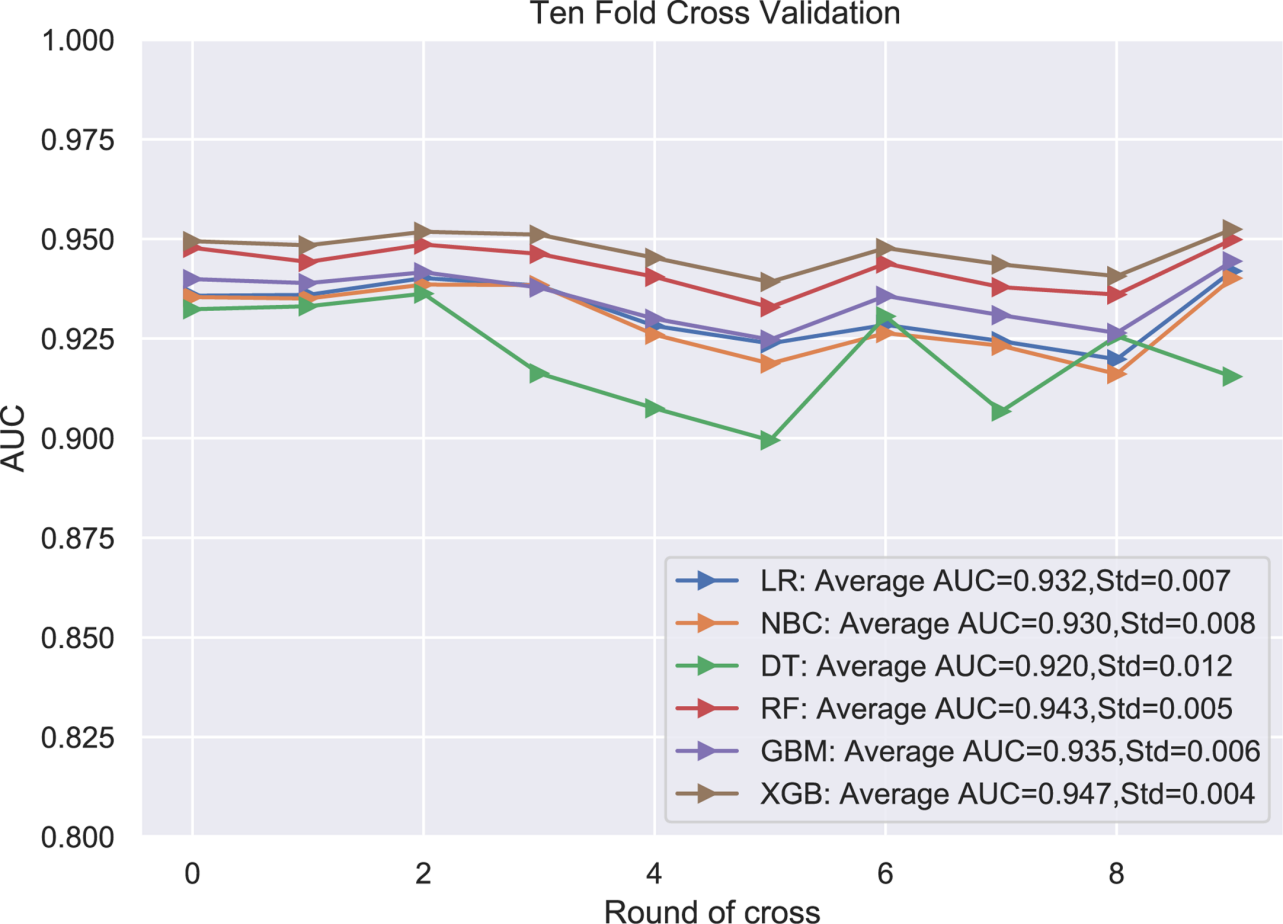
The plot of 10-fold cross-validation. LR, logistic regression; GBM, Gradient Boosting Machine; XGB, Extreme Gradient Boosting; RF, random forest; DT, decision tree; NBC, naive Bayesian model (Naive Bayes Classifier); AUC, area under the curve.

Six relevant models (LR, GBM, XGB, RF, DT, NBC) were applied to analyze the data and to select an optimal prediction model. Ten-fold cross-validation was used to compare the prediction performance of these six different ML algorithm models (**Figure 3**). As shown in **Figure 3**, all prediction models were better performed by comparing the AUC values, which were >0.9. The average AUC of XGB was 0.947, which was the highest AUC value of all predictive ML models (**Figure 3**). Therefore, the XGB model performed best and was finally selected as the preferred prediction model.

**Figure 4:**
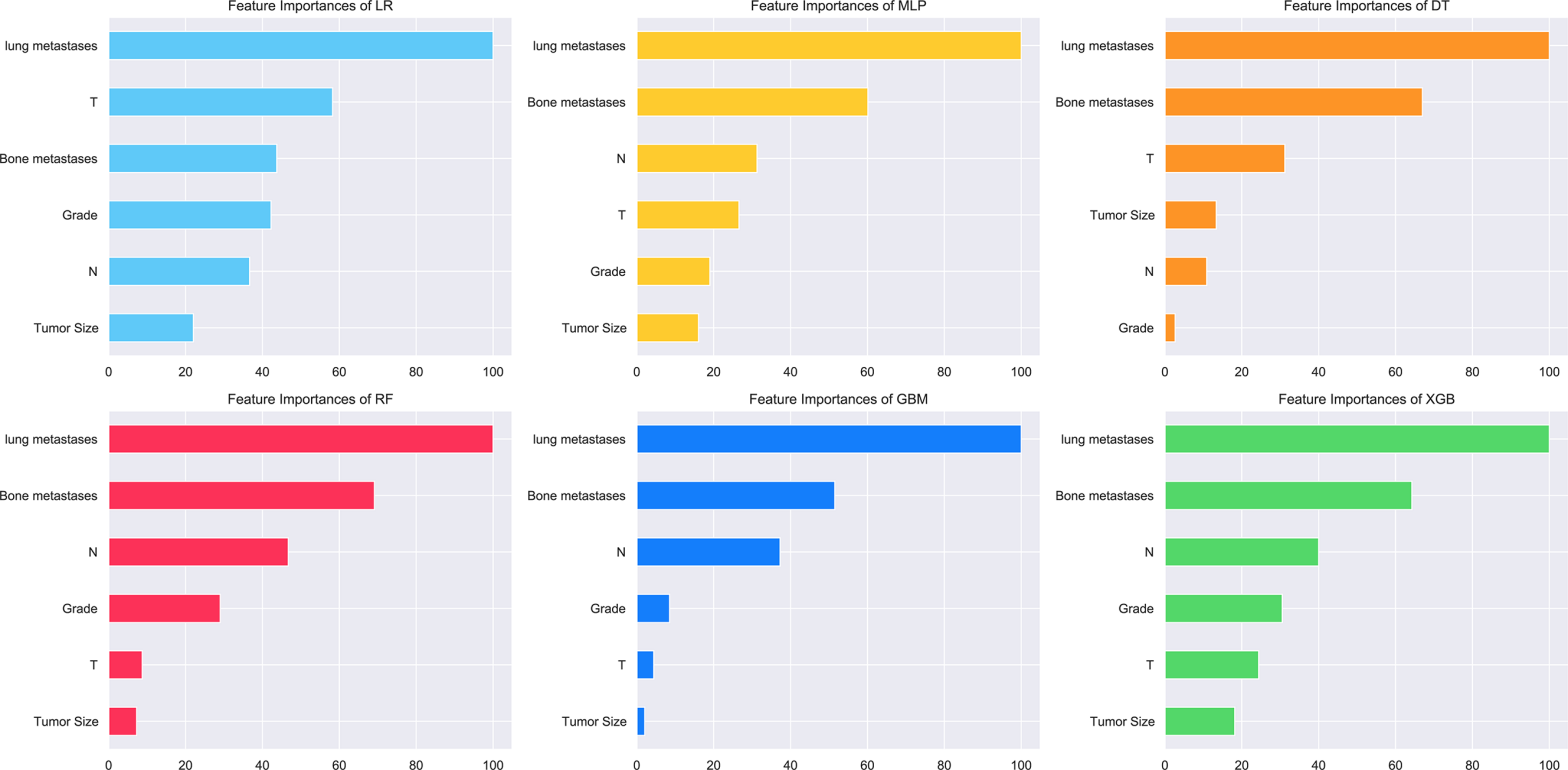
Feature importance distribution map of the six models.

The relative importance of variables in the six ML algorithms varied for the features. Lung metastasis was the most important variable in all six models, except in the DT model, while tumor size was the least important variable in the other five models. In the XGB model, the features were ranked according to their importance in the following order: lung metastasis, bone metastasis, N stage, grading, T stage, and tumor size (**Figure 4**).

### 3.4 Validation of the ML models

**Figure 5:**
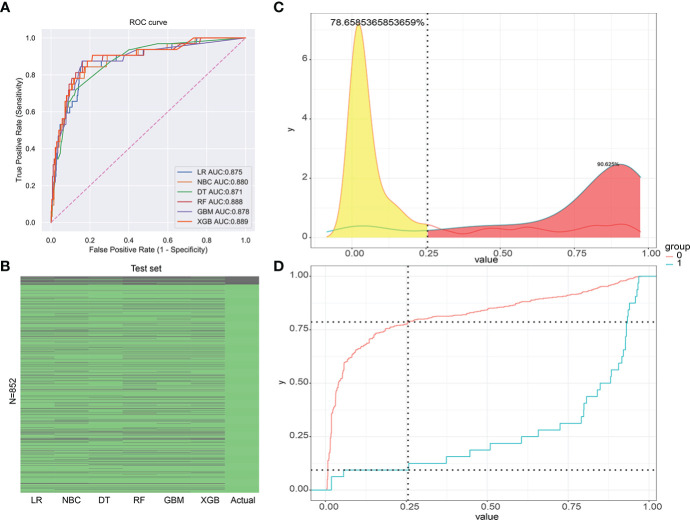
**(A)** The receiver operating characteristic curve (ROC) of the validation group (1-Specificity: false positive rate, Sensitivity: true positive rate). **(B)** The prediction of results for the validation group. **(C)** The risk density map of the model for LM (The red curve represents group 0, which means the group without LM. The blue curve represents group 1, which means the group with LM.). **(D)** The clinical utility map of the model for LM.

The validation group data were employed for validation of the training group results of the six ML models. This design increased the accuracy by comparing to univariate prediction of diagnosed RCC patients with LM. The AUC value of the XGB model was the highest (AUC = 0.889). Thus, the XGB model was the most accurate of the six models (**Figure 5A**). The XGB prediction results of the validation group showed higher accuracy compared to the actual situation than the other models (**Figure 5B**). The XGB prediction model can better distinguish RCC patients with or without LM with high efficacy (**Figures 5C, D**).

### 3.5 Construction of the web calculator

**Figure 6:**
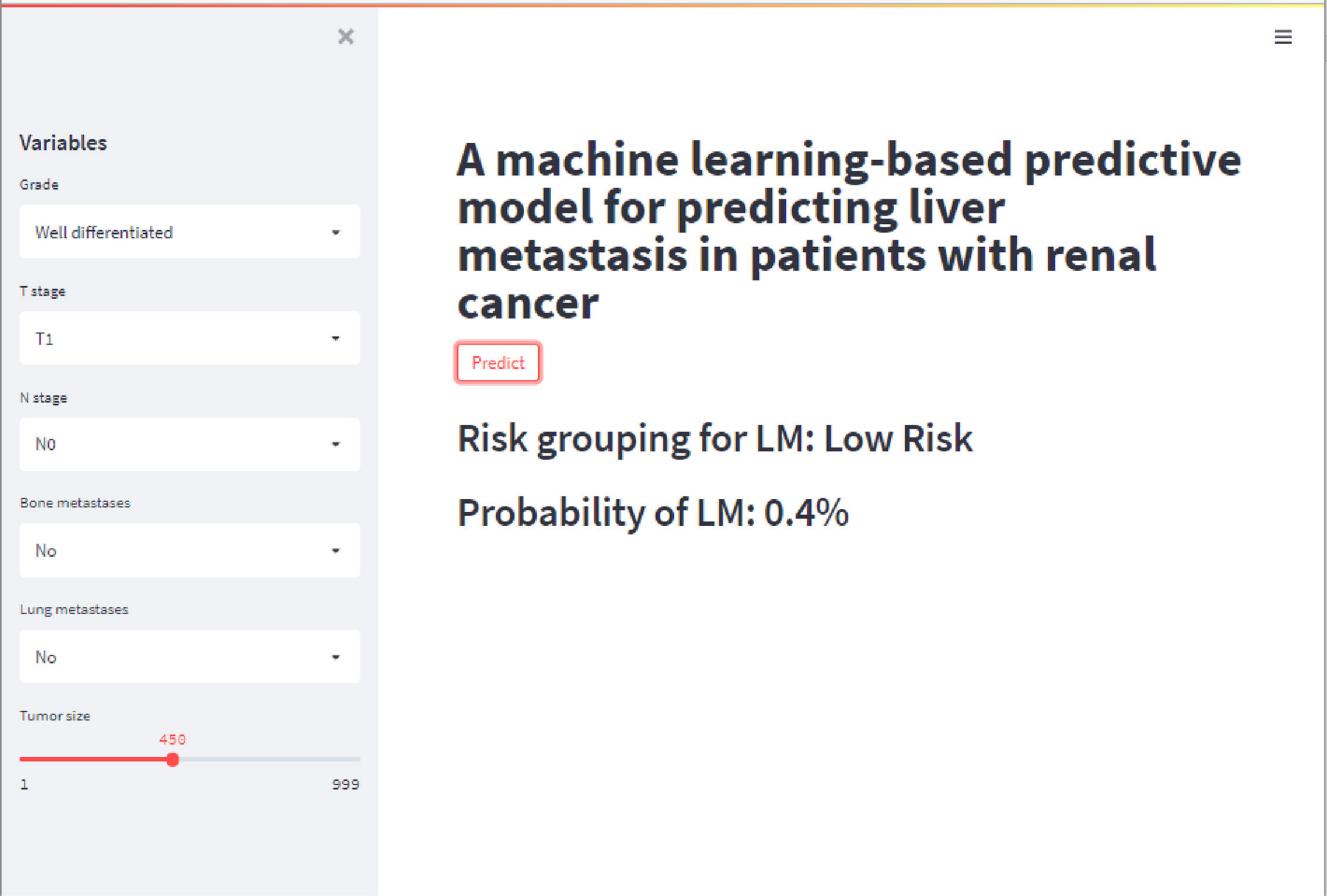
The web calculator for predicting the risk of RCC patients with LM.

In this study, a web-based online calculator was developed based on the results of the XGB model (**Figure 6**). Clinicians were able to predict the risk of developing LM in their patients by entering relevant variables and clinical features of patients with impending RCC. The operation interface was shown in **Figure 6**. The website was as follows: https://share.streamlit.io/liuwencai4/renal_liver/main/renal_liver.py (**Figure 6**).

## 4 Discussion

In 2020, new cases of RCC globally increased to approximately 430,000 and deaths to approximately 180,000 (1). RCC is a highly vascularized tumor and prone to distant metastasis (32). About 30% of new cases were metastatic at diagnosis (33). The liver is one of the most common metastatic sites of RCC, including 23.6% of newly diagnosed metastatic RCC cases (34). RCC with LM usually resulted in a poor overall survival (34). Although therapy strategies for metastatic RCC have improved significantly over the past decade, there is no consensus yet about the optimal clinical strategy for treating RCC with LM (35–37). A predictive model for RCC with LM is helpful for treatment in the clinic (38).

Regression is a statistical method for illustrating the connections between a dependent variable and two or more independent factors (38). Although statistics facilitate the understanding and interpretation of data, in recent years, ML includes algorithmic methods that enable machines to solve problems without specific computer programming, leading the way in predictive modeling tasks. It is a fast-rising field that has been widely used in the biomedical field (39). The advent of ML tools enables mining of new morphometric phenotypes and could improve patient management across a range of cancer types in the field of digital pathology (39). The International mRCC Database Consortium (IMDC) model was developed to analyze the prognosis of kidney cancer (40, 41). The Memorial Sloan Kettering Cancer Center (MSKCC) model (42), the Mayo Clinic stage, size, grade, necrosis (SSIGN) score system (43), and the modified Leibovich model (44) were reported and considered as efficient models for predicting the prognosis of RCC patients. Although more and more prediction models were used to predict the prognosis of renal cancer (45, 46), limitations were also obvious such as a lack of a comprehensive prognostic analysis of patients, and scoring methods and nomogram models were mainly statistical methods (15). ML models of RCC are mainly focused on the differentiation of molecular markers between benign and malignant renal masses (23). ML models of RCC with LM were absent (24).

In our study, multiple ML models were first employed to predict RCC with LM, and a relative network calculator was developed. In this study, 42,547 RCC patients were used to train the ML-predicted model and 852 RCC patients were used for validation. The accuracy and sensitivity of LR, GBM, XGB, RF, DT, and NBC ML models were trained and validated to predict the risk of RCC with LM. Pulmonary metastasis, bone metastasis, N stage, T stage, grade, and tumor size were found important factors in predicting LM in RCC through the LASSO regression method. Lung metastasis and bone metastasis were closely related to the occurrence of RCC with LM. Lung metastasis was the greatest effect factor on RCC with LM in this study (41, 47). These results corresponded to the prescience reported studies (48, 49). The above results suggested that the ML models for predicting the risk of RCC with LM were useful and promising.

The XGB algorithm was the most efficient model and was then used to develop an online calculator for predicting the risk of RCC with LM. The online calculator was fast and accurate in predicting the risk outcome of RCC with multiple variables. The ML model has accuracy and plausibility and clarified by 10-fold cross-validation and relevant external validations. This AI-based strategy was helpful for clinicians to choose rational treatment options. The retrospective study that excluded individual cases with incomplete data is a limitation of this study. It may cause selectivity bias, which required further validation. The online predictive calculator was helpful for clinicians to obtain predictive risks and select personalized therapeutic strategies for RCC patients with LM.

In conclusion, a predictive model for RCC with LM was constructed through ML, and a corresponding web calculator was built to assist clinicians in determining the risk of RCC with LM. By assessing the individual risk, clinicians can make appropriate interventions in advance using the ML-predicted model to prolong the survival of patients.

## 5 Conclusion

The meaningful risk factors bone metastasis, lung metastasis, grading, T stage, N stage, and tumor size were selected by LASSO regression. The LR, GBM, XGB, RF, DT, and NBC ML models were used to analyze large numbers of training group data. The XGB model was selected as the optimal prediction model with the results of 10-fold cross-validation. In the validation group, the XGB model also showed the most efficacy in predicting the risk of RCC patients with LM based on discriminant analysis. A web calculator was constructed to predict the risk factors of RCC patients with LM easily and quickly.

## Data availability statement

The original contributions presented in the study are included in the article/supplementary material, further inquiries can be directed to the corresponding authors.

## Ethics statement

Ethical review and approval was not required for the study on human participants in accordance with the local legislation and institutional requirements. Written informed consent from the participants’ legal guardian/next of kin was not required to participate in this study in accordance with the national legislation and the institutional requirements.

## Author contributions

CLY and GXZ completed the entire research design. WLL and ZYW participated in the research and collected and analyzed the data ZYW, CX and WCL drafted manuscripts. MYZ and JAZ provided expert consultation and advice. MFS,XWF, QWY and XES helped polish the language. All authors reviewed the final version of the manuscript.
